# Pre-Frailty Increases the Risk of Adverse Events in Older Patients Undergoing Cardiovascular Surgery

**DOI:** 10.5935/abc.20170131

**Published:** 2017-10

**Authors:** Miguel K. Rodrigues, Artur Marques, Denise M. L. Lobo, Iracema I. K. Umeda, Mayron F. Oliveira

**Affiliations:** 1 Hospital Sírio Libanês, São Paulo, SP - Brasil; 2 Instituto Dante Pazzanese de Cardiologia, São Paulo, SP - Brasil; 3 Faculdade Metropolitana da Grande Fortaleza (FAMETRO), Fortaleza, CE - Brasil; 4 Universidade Fortaleza (UNIFOR), Centro de Ciências da Saúde, Fortaleza, CE - Brasil

**Keywords:** Aging, Cardiovascular Surgery, Adverse Events, Fragility

## Abstract

**Background:**

Frailty is identified as a major predictor of adverse outcomes in older surgical patients. However, the outcomes in pre-frail patients after cardiovascular surgery remain unknown.

**Objective:**

To investigate the main outcomes (length of stay, mechanical ventilation time, stroke and in-hospital death) in pre-frail patients in comparison with no-frail patients after cardiovascular surgery.

**Methods:**

221 patients over 65 years old, with established diagnosis of myocardial infarction or valve disease were enrolled. Patients were evaluated by Clinical Frailty Score (CFS) before surgery and allocated into 2 groups: no-frailty (CFS 1~3) vs. pre-frailty (CFS 4) and followed up for main outcomes. For all analysis, the statistical significance was set at 5% (p < 0.05).

**Results:**

No differences were found in anthropometric and demographic data between groups (p > 0.05). Pre-frail patients showed a longer mechanical ventilation time (193 ± 37 vs. 29 ± 7 hours; p<0.05) than no-frail patients; similar results were observed for length of stay at the intensive care unit (5 ± 1 vs. 3 ± 1 days; p < 0.05) and total time of hospitalization (12 ± 5 vs. 9 ± 3 days; p < 0.05). In addition, the pre-frail group had a higher number of adverse events (stroke 8.3% vs. 3.9%; in-hospital death 21.5% vs. 7.8%; p < 0.05) with an increased risk for development stroke (OR: 2.139, 95% CI: 0.622-7.351, p = 0.001; HR: 2.763, 95%CI: 1.206-6.331, p = 0.0001) and in-hospital death (OR: 1.809, 95% CI: 1.286-2.546, p = 0.001; HR: 1.830, 95% CI: 1.476-2.269, p = 0.0001). Moreover, higher number of pre-frail patients required homecare services than no-frail patients (46.5% vs. 0%; p < 0.05).

**Conclusion:**

Patients with pre-frailty showed longer mechanical ventilation time and hospital stay with an increased risk for cardiovascular events compared with no-frail patients.

## Introduction

Frailty is characterized as a multidimensional syndrome with decline in physiologic and cognitive status.^1^ In addition, pre-frailty and frailty have been described as biological syndromes resulting from the dysregulation of multiple metabolic pathways.^1-3^

Recent data have revealed a significant association between pre-frailty and the risk of cardiovascular disease - with 25-50% more cardiovascular events in frail older individuals than in healthy elderly subjects^2^ - irrespective of any classical cardiometabolic risk factors, suggesting that pre-frailty should be targeted as a potentially reversible risk factor for cardiovascular diseases in the older population.^1^

In recent years, the number of older patients undergoing cardiovascular surgery has increased, and the number of complications from cardiovascular surgery in this population is higher compared with younger patients.^4,5^ A comprehensive preoperative assessment is essential in order to determine the risks and benefits of surgical intervention in this population; however, current methods of risk stratification have some limitations.^6,7^

Frailty has also been consistently identified as a major predictor of adverse outcomes in older surgical patients.^4,8^ Higher levels of frailty lead to increased risk during the postoperative period, with more time on mechanical ventilation, longer hospital stay, and more postoperative complications (stroke and death) compared with patients with low frailty levels.^3^ However, most studies have focused exclusively on demonstrating that patients with frailty are more susceptible to adverse events than patients without frailty after cardiovascular surgery,^8^ while the outcomes for patients in early stages of frailty (pre-frailty) are still unknown.

Therefore, we aimed to investigate the main outcomes after cardiovascular surgery in pre-frail patients compared with non-frail patients. We hypothesized that pre-frail patients have a higher incidence of cardiovascular events compared with non-frail patients. Early detection of pre-frailty enables a more careful preoperative classification of these individuals and encourages the development of prevention programs in this population.

## Methods

The present investigation was conducted as a prospective observational study. A convenience sample of 283 patients over 65 years of age were enrolled in this study. All patients had an established diagnosis of cardiovascular disease (myocardial infarction, valve regurgitation or stenosis), determined by previous electrocardiogram and/or Doppler echocardiography, and all had surgical indications (coronary artery bypass [CAB], valve replacement or valve repair, or combined surgery). Patients with prior neurological disease (previous stroke or muscular dystrophies), cognitive impairment resulting from previous injury, frailty score ≥ 5, non-elective/emergency surgery procedures and patients who refused to participate in the study were excluded.

Twenty-four hours before elective surgery, frailty of all patients was assessed by Clinical Frailty Score (CFS) (Chart 1), which was performed by a single physiotherapist, previously trained. All patients were able to participate in the assessment in an active way. Then, the patients were allocated into two groups: no-frailty (CFS 1~3) and pre-frailty (CFS 4).^9,10^ The CFS is a practical, efficient and validated scale that measures frailty. It was developed to provide clinicians with an easily applicable tool to stratify older adults according to level of vulnerability.^11^

**Chart 1:** Clinical Frailty Scale. Adapted from Rockwood9 and McDermid.10

1 Very Fit - People who are robust, active, energetic and motivated. These people commonly exercise regularly. They are among the fittest for their age.	6 Moderately Frail - People need help with all outside activities and with keeping house. Inside, they often have problems with stairs and need help with bathing and might need minimal assistance (cuing, standby) with dressing.
**2 Well** - People who have no active disease symptoms but are less fit than category 1. Often, they exercise or are very active occasionally, e.g. seasonally.	**7 Severely Frail** - Completely dependent for personal care, from whatever cause (physical or cognitive). Even so, they seem stable and not at high risk of dying (within ~ 6 months).
**3 Managing Well** - People whose medical problems are well controlled, but are not regularly active beyond routine walking.	**8 Very Severely Frail** - Completely dependent, approaching the end of life. Typically, they could not recover even from a minor illness.
**4 Vulnerable** - While not dependent on others for daily help, often symptoms limit activities. A common complaint is being “slowed up”, and/or being tired during the day.	**9 Terminally iII** - Approaching the end of life. This category applies to people with a life expectancy <6 months, who are not otherwise evidently frail.
**5 Mildly Frail** - These people often have more evident slowing, and need help in high order IADLs (finances, transportation, heavy housework, medications). Typically, mild frailty progressively impairs shopping and walking outside alone, meal preparation and housework.	

All patients were admitted to the intensive care unit (ICU) after undergoing cardiovascular surgery. Heart rate, mean arterial pressure, and oxyhemoglobin saturation by pulse oximetry (SpO_2_) were measured with a Dixtal monitor (DX 2010â), and all of them were followed up (60 days) for hospital discharge or major adverse cardiovascular events: stroke, infection and in-hospital death. In addition, length of stay, duration of mechanical ventilation, use of vasopressor agents, and the need for home-based physiotherapy services after hospital discharge were also evaluated.

The study was approved by the Institutional Ethics Committee (registration number - 1048554). Written informed consent was obtained from all participants.

### Statistical analysis

Statistical analysis was carried out using the SPSS program (version 20; SPSS Inc.). Data are expressed as mean ± standard deviation and percentage, as appropriate. The Kolmogorov-Smirnov test was used to determine normality of the data distribution; the non-paired t test and the χ^2^ test were used to assess differences in categorical data.

The survival variables were compared using the log rank test, and Kaplan-Meier survival curves were constructed. Subsequently, Cox regression models were used to assess the relationship between baseline (surgery data) frailty and mortality. Follow-up time was calculated in days from the date of the baseline measurement to the date of a major adverse cardiovascular event. The odds ratio (OR), hazard ratio (HR), and 95% confidence intervals (95% CIs) were calculated. For all of the analysis, the statistical significance was set at 5% (p < 0.05).

## Results

A total of 283 patients who underwent elective cardiovascular surgery were enrolled in this study, and of these 62 patients were excluded: 11 patients refused to participate, 17 patients had a CFS > 5, 22 patients had their post-surgery data lost, and 12 patients underwent non-elective/emergency surgical procedures. Thus, 221 patients were included in the study: 144 with pre-frailty and 77 without frailty.

Baseline characteristics are shown in Table 1. There were a higher percentage of male patients in both groups, and body mass index was slightly increased in the pre-frail group than in patients without frailty. None of the patients had heart failure or renal insufficiency prior to surgery. Moreover, there were no differences in CAB or valve replacement surgery between groups (Table 1). In addition, cardiopulmonary bypass time and cross-clamping time during procedures (extracorporeal circulation) were similar between pre-frailty and no-frailty groups (Table 1).

**Table 1:** Patients' characteristics

	No-frailty	Pre-frailty	p value
	(n = 77)	(n = 144)	
**Anthropometrics/Demographics**			
Male, n (%)	52 (67.5%)	93 (64.5%)	0.26
Age, years	70 ± 2	72 ± 4	0.42
Weight, kg	69.3 ± 9.8	73.4 ± 14.3	0.02
Height, m	1.64 ± 0.09	1.63 ± 0.10	0.76
BMI, kg/m^2^	25.4 ± 2.6	27.1 ± 3.9	0.001
LVEF, %	54 ± 12	55 ± 11	0.52
Euro Score	2 ± 0.5	6 ± 0.4	< 0.001
ASA	2 ± 0.3	3 ± 0.6	< 0.001
**Main comorbidities**			
Hypertension, n (%)	58 (75.3%)	120 (83.3%)	0.01
Type II Diabetes, n (%)	27 (35%)	56 (38.8%)	0.12
Dyslipidemia, n (%)	33 (42.8%)	66 (45.8%)	0.38
Smoker, n (%)	14 (18.2%)	16 (11.1%)	0.09
**Surgical data**			
Coronary artery bypass, n (%)	41 (53.2%)	83 (57.6%)	0.65
Valve replacement, n (%)	25 (32.4%)	42 (29.2%)	0.42
Coronary artery bypass + valve replacement, n (%)	11 (14.2%)	19 (13.2%)	0.71
Activated partial thromboplastin time, s	27 ± 6	25 ± 7	0.19
Cardiopulmonary bypass time, min	100 ± 40	90 ± 39	0.17
Cross-clamp time, min	73 ± 26	63 ± 31	0.12
**Baseline hemodynamic and blood measurements**			
HR, bpm	97 ± 22	93 ± 19	0.21
MAP, mmHg	98 ± 11	101 ± 14	0.43
Hemoglobin, g/dL	10.7 ± 2.1	10.8 ± 1.7	0.68
Hematocrit, %	33.2 ± 6.0	33.9 ± 8.7	0.49
Platelets, mm^3^	143,126 ± 60,725	146,726 ± 53,742	0.64
Creatinine, mg/dL	1.16 ± 0.50	1.27 ± 0.54	0.54
hs-CRP, mg/L	8.8 ± 0.8	9.0 ± 0.8	0.86
PaO_2_, mmHg	118 ± 5	117 ± 9	0.90
PaCO_2_, mmHg	42 ± 11	39 ± 8	0.06
HCO_3_^-^, mmol/L	22 ± 2	21 ± 3	0.53
SpO_2_, %	96 ± 4	97 ± 3	0.37

Definition of abbreviations: BMI: body mass index; LVEF: left ventricular ejection fraction; ASA: American society of anesthesiologists; HR: heart rate; MAP: mean arterial pressure; hs-CPR: high sensitive c-reactive protein; PaO_2_: arterial oxygen pressure; PaCO_2_: arterial carbon dioxide pressure; HCO_3_-: bicarbonate; SpO_2_: oxyhemoglobin saturation by pulse oximetry. Values are expressed in mean ± standard deviation or frequency. Non-paired t student test was applied to variables described as mean ± standard deviation and the χ^2^ test was used to assess differences of frequencies in categorical variables.

No differences in hemodynamic values or blood samples were observed between the groups after admission to the ICU (Table 1). However, pre-frailty group had a higher number of patients using vasopressor medications compared with no-frailty group (Table 2). A longer time in mechanical ventilation, with more patients in prolonged ventilation, as well as longer ICU and total hospital length of stay was observed in the pre-frailty group compared with the group without frailty. In addition, in the pre-frailty group, there was a higher incidence of cardiovascular events and a greater number of patients with stroke and in-hospital deaths than in the no-frailty group (Table 2).

**Table 2:** Prospective data observed at the intensive care unit and until hospital discharge in no frail and pre-frail groups

	No-frailty (n = 77)	Pre-frailty (n = 144)	p value
**Length of stay**			
Intensive care unit, days	3 ± 1	5 ± 1	0.03
Total time hospitalization, days	9 ± 3	12 ± 5	< 0.001
**Mechanical ventilation**			
Time in Mechanical ventilation, hours	29 ± 7	193 ± 37	0.001
Prolonged time in mechanical ventilation, n (%)	0	21 (14.5%)	0.001
**Vasopressor**			
Noradrenaline, n (%)	26 (33.8%)	46 (31.9%)	0.87
Dobutamine, n (%)	8 (10.4%)	29 (20.1%)	0.03
Dopamine, n (%)	14 (18.2%)	15 (10.4%)	0.08
Nitroglicerine, n (%)	8 (10.4%)	20 (13.8%)	0.24
**Adverse events**			
Infection, n (%)	4 (5.2%)	7 (4,8%)	0.69
Stroke, n (%)	3 (3.9%)	12 (8.3%)	0.02
In-hospital deaths, n (%)	6 (7.8%)	31 (21.5%)	0.001
**Home care facility**			
Physiotherapy, n (%)	0	67 (46.5%)	< 0.001

Values are expressed in mean ± standard deviation or frequency. Non-paired t student test was applied to variables described as mean ± standard deviation and the χ^2^ test was used to assess categorical data differences in frequency variables.

**Figure 1:**
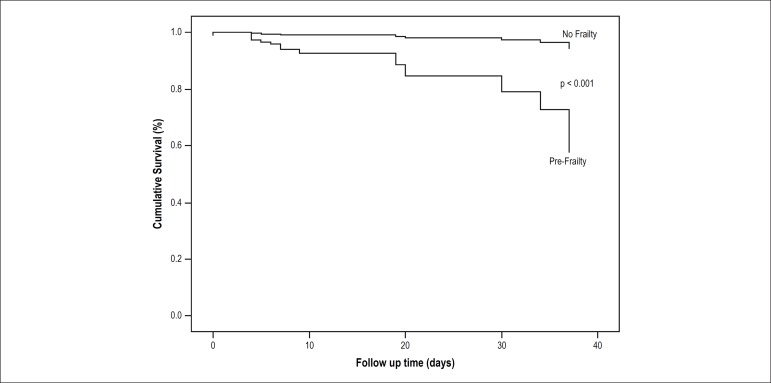
Cumulative survival of stroke events between no-frail and pre-frail groups.

**Figure 2:**
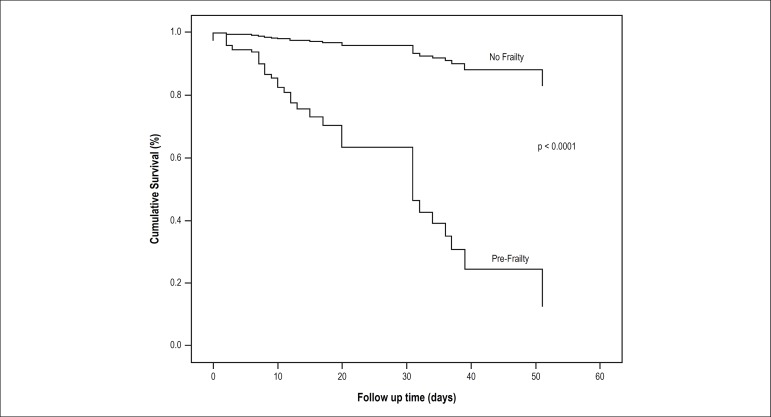
Cumulative survival of in-hospital deaths events between no-frail and pre-frail groups.

Kaplan-Meier analysis showed that cumulative events were significantly higher in patients with pre-frailty, both in stroke (Figure 1) and in-hospital deaths (Figure 2). Moreover, the OR and HR indicated an increased risk for stroke and in-hospital deaths in patients with higher frailty scores (pre-frailty group; Table 3).

**Table 3:** Odds ratio and hazard ratio for stroke and in-hospital deaths in the pre-frail group

** **	**OR**	**95%CI**	**p value**
Stroke	2.139	0.622 - 7.351	0.001
In-hospital deaths	2.763	1.206 - 6.331	0.0001
** **	**HR**	**95%CI**	**p value**
Stroke	1.809	1.286 - 2.546	0.001
In-hospital deaths	1.830	1.476 - 2.269	0.0001

OR: odds ratio; HR: hazard ratio; IC: interval of confidence.

## Discussion

In the present study, we investigated the relationship between pre-frailty and adverse postoperative outcomes following cardiovascular surgery. The main and new findings of the present study were: 1) Pre-frailty patients have more cumulative events than no-frailty patients, both in stroke and in-hospital deaths, and 2) Pre-frailty patients have longer mechanical ventilation time and hospital stay compared with the no-frailty patients. These findings are strongly relevant, as there are no previous studies that have demonstrated a relationship between pre-frailty and adverse postoperative outcomes in cardiovascular surgery.

Our results contribute to understanding whether the extent of premorbid deficit accumulation adds prognostic value in patients after cardiovascular surgery. Currently, more than half of all cardiovascular surgeries are performed on patients over 75 years^12^. A recent systematic review^13^ showed that the incidence of frailty increased steadily with age (65-69 years: 4%; 70-74 years: 7%; 75-79 years: 9%; 80-84 years: 16%; and older than 85 years: 26%), as a consequence of age-related decline in many physiological systems, which collectively results in vulnerability to sudden changes in health status triggered by minor stressor events.^2^ It also has been demonstrated that these patients have an increased risk of falls, prolonged hospitalization and mortality after surgery.^14^ Moreover, previous data have shown that each one-point increase in frailty score is associated with increased incidence of functional limitation and higher mortality risk in six months.^15^ A prospective study showed that 47% of a total cohort of 5,210 patients over 65 years of age were classified as pre-frailty (phenotype model), with an increased mortality rate (23%) during seven years of follow-up.^16^ Sundermann et al.^17^ reported that pre-frailty patients have an intermediate outcome between frailty and no-frailty patients. In addition, pre-frailty has been associated with a four-fold higher risk of becoming frail over a four-year follow-up period^16^. Sergi et al.^1^ found that patients with pre-frailty have more cardiovascular diseases compared with no-frailty patients. However, most of frailty studies on postoperative outcomes have only compared frailty versus no-frailty patients.^1,18,19^ In this context, the present study extends the knowledge regarding pre-frailty patients. Over a short follow-up period, pre-frailty patients who underwent cardiovascular surgery had more major adverse cardiovascular events and in-hospital deaths than no-frailty patients. Thus, our study presents new evidence suggesting that pre-frailty patients should be better evaluated and rehabilitated prior to cardiovascular surgery.

In our study, pre-frailty patients undergoing cardiovascular surgery had a higher incidence of stroke. In fact, this is a common finding in the scientific literature, and has been related to aging^20^ and intraoperative period, although previous studies have not evaluated frailty or pre-frailty.^21^ Actually, frailty patients undergoing non-cardiovascular surgery had more intraoperative cerebral desaturation compared with no-frailty patients,^22^ and older patients with comorbidities such as hypertension and diabetes might be at increased risk due to changes in autoregulation of cerebral blood flow.^23^ Our data are in line with current literature that suggests that pre-frailty patients undergoing a valve replacement present a higher incidence of stroke compared to patients submitted to CAB, this fact can be explained due to the higher cardiopulmonary bypass and anoxia time during surgery. Interestingly, 25% of pre-frailty patients with stroke progressed to death during the hospitalization period, demonstrating that patient's pre-morbid health status is an important point to be evaluated and may influence the prognosis after a critical event. Furthermore, they were more likely to experience cerebrovascular events and prolonged mechanical ventilation. In this context, it is highly likely that these findings explain the high incidence of stroke in the pre-frailty group in our study. Also, the higher percentage of hypertension and diabetes observed in this group might be related to an increased incidence of cerebrovascular events in these patients.

It is well established that prolonged mechanical ventilation has been related to new deficits or worsening of pre-existing deficits associated with the frailty syndrome in critically ill patients, that persist even after resolution of the critical condition,^24^ regardless of the use of invasive or non-invasive ventilation.^15,25^ Our patients with pre-frailty had higher mechanical ventilation time. In fact, increased mechanical ventilation time might be a consequence of the main complications found in our study.

Moreover, prolonged mechanical ventilation is associated with impaired functionality, longer hospitalization period and higher incidence of in-hospital deaths.^26^ It has been demonstrated that more than 80% of these patients require a second hospitalization within 12 months of discharge from the ICU^26^ with high incidence of six-month mortality.^27,28^ Furthermore, those patients who survive may have worsened functional capacity for almost five years after hospital discharge.^29^ Although it is out of scope of our study to follow up patients after hospital discharge, the pre-frailty group had longer hospital length of stay, required treatment in skilled or assisted-living facility, including physiotherapy and rehabilitation after discharge. Together, these findings suggest that this group is on increased risk of re-hospitalization and/or death in a short period of time.

### Clinical implications

Frailty is recognized as a multi-dimensional syndrome characterized by the loss of reserves (physical and cognitive) that result in vulnerability. The CFS is an easy-to-use frailty scale for risk stratification of older adults that enables the assessment of frailty-related outcomes even in the preoperative period, and may improve treatments and interventions, prevent possible complications, and reduce the length of stay.

Our study presents important clinical findings, as frailty is a reversible condition when treated in the early stages with interventions such as exercise. These interventions are effective and might delay the transition from pre-frailty to frailty.^30^ Exercise prior to cardiovascular surgery may also contribute to better recovery in ICU.

In addition, our study emphasizes the need to incorporate a frailty evaluation before cardiovascular surgery, in order to better understand the risks to these older patients and to guide specific interventions in the preoperative period to minimize the risk of adverse events, even in patients in the early stages of frailty.

### Study limitations

This study has some limitations that should be addressed. There is a blank in the recent literature regarding the best evaluation criteria for frailty. There is significant heterogeneity among frailty criteria in clinical trials, thus making it more difficult to recognize and identify frailty in post-surgical patients.^31^

Pre-frailty group had a larger number of patients than no-frailty group. To rule out the possibility that this issue might affect our findings, statistical power for the main outcomes was calculated and revealed a power of 99.98% for total time hospitalization and 74.22% for in-hospital death.

A recent study showed that widely used scores (Acute Physiology Score and Acute Physiology and Chronic Health Evaluation) failed to predict higher death risk.^32^ However, frailty, when associated to traditional risk scales (ASA, Eagle and Lee), is an independently predictor of postoperative complications, length of stay, and requirement of skilled or assisted-living care after hospital discharge in older surgical patients.^8^ Our study took care to evaluate some types of risk scale: CFS, ASA and EuroScore, and all of them were increased in patients that had worse outcomes. Furthermore, frailty was able to predict major cardiovascular events in post-cardiac surgery, even in patients with early stages of frailty.

There are two frailty models: phenotype and cumulative deficit models. We decided to use just the CFS because it is readily available at the bedside and is easier to understand and use than other frailty assessment tools. Moreover, the CFS has been considered an optimal tool for use on admission to the ICU.^10^

## Conclusion

Patients with pre-frailty showed longer mechanical ventilation time, longer ICU and hospital length of stay, and higher requirement for home-based physiotherapy services than no-frailty patients after cardiovascular surgery. Moreover, the presence of pre-frailty on pre-operative period predicts more cumulative events (stroke or in-hospital death). However, it remains unknown whether pre-frailty treatment before cardiovascular surgery is effective to prevent cumulative outcomes.
